# Baseline Immune Activity Is Associated with Date Rather than with Moult Stage in the Arctic-Breeding Barnacle Goose (*Branta leucopsis*)

**DOI:** 10.1371/journal.pone.0114812

**Published:** 2014-12-17

**Authors:** Cecilia A. M. Sandström, Jouke Prop, Henk van der Jeugd, Maarten J. J. E. Loonen

**Affiliations:** 1 University of Groningen, Arctic Centre, PO Box 716, 9700 AS Groningen, the Netherlands; 2 University of Groningen, Ocean Ecosystems, Nijenborgh 7, 9747 AG Groningen, the Netherlands; 3 Vogeltrekstation - Dutch Centre for Avian Migration and Demography (NIOO-KNAW), PO Box 50, 6700 AB Wageningen, the Netherlands; CEA, France

## Abstract

Variation in immune defence in birds is often explained either by external factors such as food availability and disease pressure or by internal factors such as moult and reproductive effort. We explored these factors together in one sampling design by measuring immune activity over the time frame of the moulting period of Arctic-breeding barnacle geese (*Branta leucopsis*). We assessed baseline innate immunity by measuring levels of complement-mediated lysis and natural antibody-mediated agglutination together with total and differential leukocyte counts. Variation in immune activity during moult was strongly associated with calendar date and to a smaller degree with the growth stage of wing feathers. We suggest that the association with calendar date reflected temporal changes in the external environment. This environmental factor was further explored by comparing the immune activity of geese in the Arctic population with conspecifics in the temperate climate zone at comparable moult stages. In the Arctic environment, which has a lower expected disease load, geese exhibited significantly lower values of complement-mediated lysis, their blood contained fewer leukocytes, and levels of phagocytic cells and reactive leukocytes were relatively low. This suggests that lower baseline immune activity could be associated with lower disease pressure. We conclude that in our study species, external factors such as food availability and disease pressure have a greater effect on temporal variation of baseline immune activity than internal factors such as moult stage.

## Introduction

Energy investment in the immune system can be viewed as a trade-off between the costs and benefits of maintaining an active system. Costs are associated with the energy and nutrients needed for production of components, unnecessary expenditure of energy due to misregulation, and the running and maintenance of the immune system [1]–[3]. Additionally, any resources channelled towards the immune system will not be available for other demanding processes [4]–[6]. As a consequence, relative costs are high when investment in competing life-history events like feather moult or reproduction, is at a premium. The benefits of an active system, and associated enhancement of disease resistance, depend strongly on environmental factors. Firstly, disease pressure can evoke a response [7] so when disease risk is low, an immune response might be unnecessary and better avoided (e.g. [8]–[10]). Secondly, food conditions limit available energy that could be spent on either moult/reproduction or immune activity [11]–[13]. When disease risk is low and food availability is high, baseline immune activity is expected to be low, and investment can be directed towards somatic growth and reproduction (or another life-history event). Thus, both the state of an individual and environmental conditions affect the activity of the immune system [14]–[17].

Determining temporal variation in activity of the immune system within a given stage of the annual cycle sheds light on (individual) flexibility and, consequently, can be used to calibrate the single time-point measurements often used in wild populations. Hegemann et al. [18] revealed patterns over the annual cycle from repeated measures on single individuals, which were consistent with patterns at the population level. In this study, we explored variation of baseline immune activity within a single period of the annual cycle. Using multiple measurements over time, we studied changes in immune activity over the period of wing feather moult. This provides a reference for baseline levels at various time-points for later studies where sampling is only possible once during the moult season. As wild animals serve both as reservoirs and sentinels of diseases [19], understanding immunological variation can predict when individuals are susceptible to infections and when further transmission of diseases is likely to occur [20], [21]. The current study focuses on explaining variation in baseline immune activity during moult, using Arctic-breeding barnacle geese (*Branta leucopsis*) as a model species.

The moulting period is an energy-demanding period due to the direct energetic costs associated with the synchronised growth of the wing feathers [22]–[25]. In addition, various indirect costs add to the stress by a more expensive metabolism: more energy is needed for thermoregulation due to temporary loss of insulating feathers [23], [26], and there are shifts in somatic tissues towards strongly developed leg muscles during the flightless period to cope with increased predator vulnerability [27]. Breeding in the energetically expensive Arctic, however, is rewarded by nutritious (though scarce) food plants [25], [28], and disease pressure from the environment may be lower than at southerly sites [29]–[32]. Reduced disease pressure in the extreme environment of the Arctic is largely due to the climate, which is inhospitable for micro- and macro-parasites and for parasite-transmitting organisms [20], [29]–[35]. For animals living in the Arctic, the inferred low disease pressure could allow a less active immune system [7]–[10], [14], [35]. Under these conditions, variation in immunity could well be connected to other proximate causes, such as those set by moult requirements.

We explored baseline innate immune activity due to its broad benefits: it is effective in controlling multiple pathogen types, provides first-line defence, responds immediately to threats, does not require previous exposure to a particular antigen, is constantly maintained and thereby predicted to generate continuous energetic costs [36]–[39]. Baseline innate levels vary over time in response to changes in environmental conditions [18], [37], which is in contrast to induced levels [40]. In this study, two categories of baseline immunity were addressed, which cover a range of protective mechanisms. Firstly, leukocyte (white blood cell) concentrations were determined, as well as the relative contributions of various leukocyte types, which provide information on circulating immune cells. These measures can be used as an index of health, reflecting innate and adaptive components of the immune system [41]. Secondly, levels of complement and natural antibodies were determined. These components of the immune system provide a line of defence against infection via cell lysis, and link the innate with adaptive immunity [38], [39].

By investigating temporal changes in baseline immune activity, we aimed to distinguish the influence of internal and external factors on the immune system. We hypothesize that if baseline immunity is mainly affected by internal factors (such as moult), then immune functions will be strongly associated with the stage of feather growth. “Moult stage” incorporates energetic costs due to restricted mobility, increased dermal inflammation due to breaking feather follicles [42], [43], and use of resources directed towards feather growth. As these stress factors are likely highest during the first days of moult, we expect immune activity to decrease over the progressive stages of moult.

Alternatively, if immune activities are mainly affected by external factors, such as disease pressure and food resources, we expect immune functions to be associated with a measure of phenological events, as approximated by calendar date. Geese are constrained in mobility during the moult period and local food resources are depleted as the season progresses [25], potentially leading to a drop in immune activity over time. Similarly, as the birds intensively graze the tundra and repeatedly use the same stretches, the risk of cross infection is likely to increase. If true, immune activities among individuals would be synchronised and there should be a strong association between immune activity and calendar date.

To further explore the aspect of environmental factors, we compared immune performance of an Arctic-breeding population of barnacle geese with the performance of barnacle geese breeding in a temperate (more southerly) environment. If immune activities are largely determined by environmental factors (including disease pressure), then we expect immune activities to be lower in the population in the Arctic than in the temperate population.

## Material and Methods

### Study populations and study areas

Barnacle geese have both migratory Arctic and sedentary temperate breeding populations. The Arctic study population breeds at Nordenskiöldkysten, Spitsbergen, Svalbard, Norway (78°N/13°E), and undertakes a 3000 km migration to the wintering area at the Solway Firth, United Kingdom (55°N/4°W) [25]. Incubation spans the period from early June through mid-July, followed by a flightless period from mid-July to mid-August when the wing feathers moult. During the moulting period, goose density at the foraging grounds is on average 10 geese per ha [44].

The temperate study population spends the summer breeding and moulting at Krammerse Slikken, the Netherlands (51°N/4°E) [45], [46]. These geese are part of a population that originated from migratory Arctic geese that ceased migration. Since the 1980s the population size has rapidly increased. The relatively young temperate population is genetically differentiated from the Arctic-breeding populations, although the difference in genetic structure is small due to a high rate of genetic exchange [47]. Incubation and moulting periods in the temperate population cover April through mid-June and early June to mid-July, respectively [46]. Goose densities during moult are on average 13 birds per ha (derived from [48]).

### Catching and field sampling

In the summers of 2007 and 2008, we captured moulting, and consequently flightless, geese. The catching period at Svalbard covered 14 days (205–218 Julian date  = 23 July until 5 August), while the catching period in the Netherlands was only four days (186–189 Julian date  = 4–8 July). Geese were herded into a V-positioned net that ended in a corral where they were collected. To minimise disturbance, geese were transferred to small tents immediately after catching. Approximately one hour after catching, measuring and sampling were initiated. Geese were individually marked by leg rings, their sex was established by cloacal examination [49], and their age group was noted as juvenile or adult (including individuals in their second calendar year). Only data collected from adults were used in subsequent analyses.

Immune measures may respond to stress caused by capture [50]. To account for potential effects of the duration that geese had been kept in captivity, the sequential order of handling was noted. As a measure of the progress of moult, we used a standard technique (e.g. [25]) by measuring the length of the longest primary feather (P9) to the nearest mm. This was done by inserting a thin ruler between the 8^th^ and 9^th^ primary and measuring the distance from the skin surface to the distal end of the feather [51]. As primaries and secondaries are shed almost simultaneously in geese and regrowth of the feathers is synchronous [23], measuring a single wing feather adequately describes moult stage. Moult of other feathers seems less of an energetic burden to geese. Moulting of body and tail feathers starts after completion of the wing moult and lasts for several months [52].

**Figure 1:**
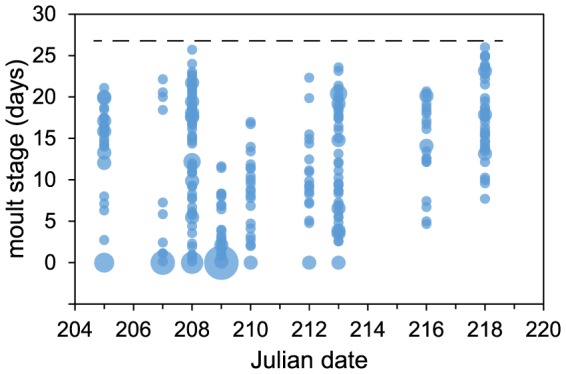
Moult stage of geese during each of the catches. Throughout the season, geese exhibited a large variation in moult stage. At later dates, some of the early moulters might have regained flight capacities (birds were able to fly after 27 moult days, as indicated by the dashed line). Size of symbols varies with sample size (1–12).

For each sampling date, we aimed to cover the whole range of moult stages present at that moment in the goose flocks. This was possible due to the strong cohesion among group members, and in most cases all individuals present were caught during a single catch. Variation in moult stage within each of the groups was high (Fig. 1), which enabled us to separate the effects of moult stage and date.

A blood sample of 0.2–2 ml was drawn from the brachial vein using a non-heparinised syringe and needle (2007: n = 114 and 50; 2008: n = 224 and 116, from the Arctic and the temperate populations, respectively). A blood smear was made [41] with a drop of fresh blood, which was air-dried and stored for later processing. Not all samples were analysed for each of the immune measures.

### Processing of blood samples

Blood samples were transported from the sampling location to a field laboratory where they were stored at cool temperatures (5°C). Samples were allowed to coagulate for 24 hours followed by centrifuging (7000 rpm, 12 min) in order to separate red blood cells (rbc) from serum. The centrifuge was a ZipSpin, 12V DC (LW Scientific) suitable for use under field conditions. The serum was subsequently stored in a mobile freezer (Waeco CoolFreeze CF-35) at −18°C for 2–3 weeks (for one week in the temperate area), then transported to the main laboratory by cold-chain, after which samples were stored at −20°C until analysis. Handling and transport of samples was similar in the Arctic and temperate study areas, though it inevitably took longer before the Arctic samples reached the final laboratory. There is little reason, however, to believe that the Arctic samples were degraded by the longer period of transport (see Discussion).

### Immunological measures

#### Leukocyte counts

After fixing in ethanol, blood smears were stained by Hemacolor and covered by cover slips embedded in Pertex. Microscope examination was at 1250× magnification for leukocyte enumeration, following a grid system covering the slide. Counts of different types of leukocytes were performed by one observer. For leukocyte identification, counts up to and including the row containing the 100^th^ leukocyte were completed, which resulted in, on average, 120 (±27, SD) identifications per slide [53]. The leukocyte density was determined by counting the numbers of leukocytes and red blood cells in 30 vision fields at 500× magnification (totalling approximately 5000 red blood cells).

Leukocytes were classified as heterophils, eosinophils, basophils, monocytes (activated or non-activated) and lymphocytes (reactive or non-reactive). Heterophils, eosinophils, basophils and monocytes are phagocytic cells, and fight infections by engulfing (phagocytising) foreign particles and removing dead or dying cells [41]. Basophils only occurred at a frequency of 0.1% and were not considered in further analyses. We distinguished activated monocytes from non-activated cells by their darker cytoplasm and coarse nuclear chromatin [53]. Activated monocytes work together with reactive lymphocytes to clear infected host cells [54]. Reactive lymphocytes were recognised by their large size and dark blue cytoplasm, which is thought to be caused by antigenic stimulation of resting lymphocytes [41]. Activated monocytes and reactive lymphocytes were grouped into the category “reactive leukocytes”. Eosinophils were grouped together with non-activated monocytes as “eos+monoc”. Heterophils are the most abundant phagocytic cell and are recognized by their orange-red, rod-shaped granules. Heterophils and lymphocytes together form the majority of leukocytes [41], [53]. The H/L-ratio, the ratio of heterophil to lymphocyte occurrence, is commonly regarded as a measure of physiological and/or social stress [53], [55].

#### Hemagglutination – Hemolysis assay

Two immunological tests were performed on the preserved serum. Firstly, solutions from a serial dilution of plasma samples (25 µl) were incubated in a fixed volume of red blood cells collected from rabbits (Harlan, UK) following the method described by Matson et al. [39]. Agglutination was scored as the negative logarithm (with base two) of the last dilution for which hemagglutination was exhibited. Hemagglutination results from the activity of natural antibodies, which causes clumping of foreign red blood cells into a pellet-like structure (agglutination). Secondly, serial dilution of serum and subsequent scoring of hemolysis were performed in the same way as for hemagglutination [39]. Hemolysis reflects the interaction of natural antibodies and complementary proteins to dissolve (i.e. lyse) foreign red blood cells. All samples were blindly scored by one observer.

### Progress of moult

Moult stage was estimated by dividing the length of the 9^th^ primary by their daily growth rate. Growth rates for the Svalbard population were 7.3 mm/day for males and 7.0 mm/day for females [56] (males are somewhat larger than females). Growth rates were presumed to be similar in the temperate population [46]. Date of initiation of moult was calculated as date of catching minus the progress of moult.

### Statistics

Whenever possible, immune measures were transformed to achieve normal distributions. Proportions were arcsine-transformed, and relative densities of leukocytes and H/L- ratios were log-transformed (base 10). Distributions of the transformed values were checked visually by Q–Q plots and were found to be reasonably close to normality (Shapiro–Wilk's statistic>0.96). Only the proportions of reactive leukocytes were not successfully transformed due to an overrepresentation of zero values (see below). Lysis and agglutination scores were composed of discrete values, which are best described by a Poisson distribution.

To explore the variation in the immune measures, the metrics were analysed with the following independent variables: year (2007 – as reference factor – or 2008) and sex (female – as a reference factor – or male) as fixed factors; Julian date, moult stage (number of days), initiation of moult (date) and order of handling as covariates. To account for non-linear trends in time, a quadratic term of Julian date was also included as an independent variables. A set of candidate models was defined containing all possible combinations of main terms explored. As the three date measures were confounded (see above), models contained no more than two of these measures (Julian date or Julian date squared, and moult stage).

Depending on the distribution of the dependent variable, relationships were analysed by linear regression (arcsine-transformed proportions, log values of leukocyte densities and H/L-ratio; lm in R), generalised linear models with a Poisson-link (lysis and agglutination; glm in R), or generalised linear models that account for distributions with excess zeros (proportions of reactive leukocytes; zeroinfl in the package pscl in R), with a Poisson distribution for the counts and a logit-link for the excess zeros [57]. For this last analysis, proportions were expressed as percentages rounded to the nearest integer.

Model selection was based on the Akaike Information Criterion corrected for finite sample sizes (AICc), as executed by the function aictab (package AICcmodavg). Selected models were those that had the lowest AICc in the candidate set. The significance of each term in the top-rated models was tested by ANOVA (comparing the models with and without the term), and terms were dropped from the final model when not significant. The relative importance of the independent variables was further assessed by calculating a cumulative AICc weight for each variable by summing the weights from all models in the candidate set that contained the variable of interest [58]. Descriptive statistics and correlation matrices of dependent and independent variables are given in the Supporting Information (S1 Table, S2 Table, S1 Figure).

#### Arctic compared to lower latitudes

Barnacle geese at temperate grounds breed and moult earlier in the season than geese at Arctic grounds [46], [56]. Although the birds were sampled when at similar moult stages (P9 mean ± SD; Arctic: 82.0 mm±51.2; temperate: 82.9 mm±43.2; t_326_ = −0.15, p = 0.88), the average sampling date differed by 23 days. To compare immune measures obtained from the two populations, a common time scale was created based on time from average initiation of moult. Mean initiation of moult for the temperate population was estimated at 176 Julian date (25 June) and 199 Julian date (18 July) for the Arctic population. Corresponding sampling occasions in both populations were day 10–14 after average initiation of moult (Julian date 186–189 and 209–212 for temperate and Arctic, respectively).

As the temperate population was sampled within a restricted period of time, testing for date effects within this population was not possible. Therefore, analysis of the difference between populations followed a similar model structure as described for the Arctic population alone but without date parameters (Julian date). Modelling started with the final model generated for the Arctic population with population (Arctic or temperate) included as a fixed factor. Non-significant terms (as derived from ANOVAs comparing models with and without the term) were omitted from the models. Analyses were performed using the statistical program R version 3.0.2 [59].

### Ethics statement

This study involved sampling and handling of the non-endangered protected species *Branta leucopsis*, which has an IUCN classification of *least concern*. The full sampling protocol was approved by Animal Welfare Officers: under licence DEC 4772A (Institutional Animal Care and Use Committee) from the University of Groningen and by *Ontheffing Flora- en Faunawet* (FF/75A/2007/032) in the Netherlands; and under licence FOTS 2767 (*Forsøksdyrutvalget*, Norwegian Animal Research Authority), by the Norwegian Food Safety Authority and by the Governor of Svalbard in Norway. Permits for catching and ringing geese in Svalbard were issued by The Norwegian Directorate for Nature Management (Terje Bø) and *Ringmerkingssentralen*, Stavanger Museum (Alf Tore Mjøs). Land access in the Netherlands (51°40′N, 4°14′E) was approved by Evert Dolman, *Staatsbosbeheer*, and in Norway (77°50′N, 13°45′E) by Ian Gjertz and Tor Punsvik, both at the Office of the Governor of Svalbard.

## Results

### Temporal variation

In the Arctic, immune activities during the moulting period were closely associated with time parameters. Based on the top-ranking models, variation was associated with Julian date in seven of the eight immune measures and with moult stage in one immune measure (proportion of eos+monoc; Table 1). Agglutination was the only measure exhibiting no (significant) association with a date parameter at all. Considering all models within 2 AIC-units of the top-ranking model (S3 Table) gave support for moult stage as an explanatory variable in another three measures (density leukocytes, proportions of heterophils and reactive leukocytes). The support for Julian date and moult stage as determinants of time variation was confirmed when considering the cumulative AIC weights of the independent variables in the complete sets of candidate models (Table 2). The explanatory power of Julian date (and its squared value) exceeded that of moult stage by a factor of 2–4 (the ratio of cumulative AICc weights of Julian date and moult stage in Table 2). The exception was the proportions of eos+monoc, which were more likely to be affected by moult stage than by Julian date.

**Table 1:** Parameter estimates based on top-ranking models.

Immune measure	Independent variable	Mean	SE	Test statistic	P
Density leukocytes (n/1000 rbc)					
	Intercept	126.13	47.64	2.65	<0.01
	Date	−1.185	0.453	−2.62	<0.01
	Date^∧^2	0.003	0.001	2.61	<0.01
	Year	−0.093	0.027	−3.43	<0.001
	Sex	−0.058	0.024	−2.43	<0.02
Lymphocytes (proportion)					
	Intercept	5.35	1.22	4.35	<0.001
	Date	−0.020	0.006	−3.39	<0.001
	Sex	−0.106	0.039	−2.69	<0.01
	Order of sampling	−0.005	0.001	−4.40	<0.001
Heterophils (proportion)					
	Intercept	−3.13	1.24	−2.52	<0.05
	Date	0.024	0.006	4.02	<0.001
	Sex	−0.106	0.040	−2.68	<0.01
	Order of sampling	0.005	0.001	5.08	<0.001
H/L-ratio					
	Intercept	−4.25	1.41	−3.01	<0.005
	Date	0.022	0.007	3.23	<0.001
	Sex	0.112	0.045	2.50	<0.05
	Order of sampling	0.005	0.001	4.41	<0.001
Eosinophils+monocytes (proportion)					
	Intercept	84.73	42.66	1.99	<0.05
	Date	−0.800	0.404	−1.97	0.05
	Date^∧^2	0.002	0.001	1.96	0.05
	Moult stage	−0.006	0.002	−3.88	<0.001
	Year	0.062	0.026	2.43	<0.05
Reactive leukocytes (proportion)					
	Intercept	−1473.0	507.6	−2.90	<0.005
	Date	13.870	4.801	2.89	<0.005
	Date^∧^2	−0.033	0.011	−2.88	<0.005
	Year	−1.336	0.243	−5.50	<0.001
Lysis (titre)					
	Intercept	−518.4	150.1	−3.45	<0.001
	Date	4.905	1.420	3.45	<0.001
	Date^∧^2	−0.012	0.003	−3.45	<0.001
	Year	0.652	0.114	5.71	<0.001
Agglutination (titre)					
	Intercept	1.74	0.05	39.29	<0.001

Parameter estimates for each of the immune measures, based on top-ranking models (as listed in S3 Table). Individual parameters that were not significant in the top-ranking models were omitted. Immune measures were transformed before analyses. The test statistic is the t-value or the z-score (for reactive leukocytes, lysis and agglutination).

**Table 2:** Cumulative AIC values of each independent variable.

Immune measure	Independent variable					
	Julian date	Julian date squared	Moult stage	Year	Sex	Order of sampling
Log density leukocytes	0.83	0.77	0.25	0.92	0.88	0.53
Lymphocytes (proportion)	0.98	0.31	0.26	0.27	0.93	0.98
Heterophils (proportion)	0.97	0.25	0.34	0.27	0.92	0.97
Log H/L-ratio	0.98	0.31	0.25	0.27	0.87	0.99
Eos+monoc (proportion)	0.62	0.44	0.96	0.93	0.36	0.45
Reactive leukocytes (proportion)	0.99	0.98	0.51	0.99	0.24	0.41
Lysis (titre)	0.97	0.97	0.25	0.97	0.36	0.25
Agglutination (titre)	0.65	0.37	0.34	0.50	0.29	0.50

Cumulative AICc values of each independent variable based on its contribution to the AICc values of the candidate model set. Values are based on the contribution of the independent variable to the AICc of the candidate model set. Full support would be indicated by 1.00.

**Figure 2:**
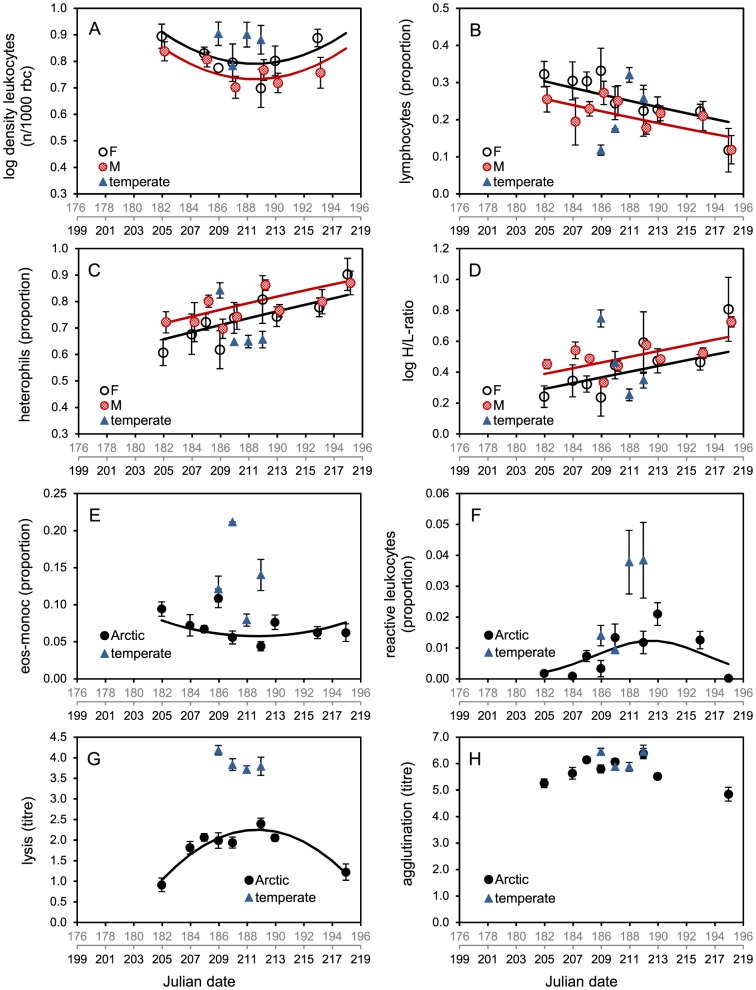
Measures of baseline immune activity (means ± SE) over time. Immune activity over the moulting season presented for each immune measure: (A) leukocyte density, (B) proportion lymphocytes, (C) proportion heterophils, (D) the H/L-ratio, (E) proportion eosinophils + monocytes (eos+monoc), (F) proportion reactive leukocytes, (G) lysis and (H) agglutination. The two x-axes show Julian dates (JD) from the average initiation of wing moult per population: the temperate population started at 176 JD (grey scale) and the Arctic population started at 199 JD (black). Males and females are displayed separately when sexes differed significantly (Table 2), otherwise means are for all birds. Trend-lines are based on the models in Table 2 (after back-transformation). The four catches in the temperate area (triangles) are too close in time to show seasonal effects.

Parameter estimates indicated that the temporal variation was different among some of the immune measures (Table 1, Fig. 2A–G). The proportion of reactive leukocytes and lytic activity showed a convex polynomial relationship with highest values halfway through the study period (Fig. 2F, G). Density of leukocytes and proportion of eos+monoc showed a concave polynomial relationship with lowest values halfway through the study period (Fig. 2A, E). Proportion of lymphocytes decreased with date (Fig. 2B), whereas proportion of heterophils and H/L-ratio increased with date (Fig. 2C, D).

### Variation between sexes

Differences in immune activity between the sexes were found in total leukocyte density, and in the closely correlated proportion of lymphocytes, proportion of heterophils and the H/L-ratio (Table 1). Females displayed more leukocytes and lymphocytes than males, whereas males had higher proportions of heterophils and higher H/L-ratios (Fig. 2A–D). None of the interaction terms between sex and date were significant (P>0.05), indicating that the changes over time were similar for males and females.

### Variation between years

Several immune parameters varied with year (Table 1). Density of leucocytes and proportion of reactive leucocytes were higher in 2007, while proportion of eos+monoc and lytic activity were higher in 2008.

### Variation by order of sampling

Proportions of lymphocytes and heterophils, and the H/L-ratio were associated with order of sampling. Proportion of lymphocytes was negatively related to the order of sampling, and the two other measures were positively related.

### Comparison with lower latitude

Compared to the Arctic population, the temperate population had higher density of leukocytes, proportion eos+monoc, proportion of reactive leukocytes and lytic activity (Table 3; also Fig. 2). Other measures did not differ between the two populations.

**Table 3:** Comparing immune measures between Arctic and temperate populations.

Immune measure	Arctic			Temperate			Test statistic	P-value	Other terms in model
	Mean	SE	N	Mean	SE	N			
Log density leukocytes	0.793	0.026	40	0.891	0.027	70	2.36	0.02	–
Lymphocytes (proportion)	0.250	0.019	60	0.242	0.015	67	0.31	NS^1^	Order, order×pop
Heterophils (proportion)	0.680	0.021	60	0.646	0.016	67	1.41	NS	–
Log H/L-ratio	0.493	0.048	60	0.488	0.043	67	0.07	NS^1^	Order, order×pop
Eos+monoc (proportion)	0.069	0.007	60	0.109	0.009	67	4.33	<0.0001	Year, moult stage
Reactive leukocytes (proportion)	0.009	0.002	60	0.031	0.006	67	5.05	<0.0001	–
Lysis (titre)	1.980	0.141	53	3.910	0.070	79	5.51	<0.0001	–
Agglutination (titre)	6.000	0.093	53	6.080	0.090	79	0.17	NS	–

Given are mean, standard error (SE), sample size (N) for the Arctic and temperate populations. Data are selected for four dates corresponding in moult stage. Initial models were those listed in Table 1, with population (pop) as an additional term. Non-significant terms were omitted. Test statistics given are t-value for the first five measures and z-value for the last three. NS = non-significant (P>0.05).

1 Difference between populations evaluated for an average value of the covariate “order”.

## Discussion

We found that immune activity during moult in Arctic migratory geese was strongly associated with calendar date, whereas moult stage was less influential. Moreover, immune activities were generally lower in the Arctic study population than in temperate areas, where both disease pressure and food abundance is predicted to be higher. Our observations support the hypothesis that changes in immune activities are strongly determined by external factors, rather than by internal factors alone. Effects of environmental factors, such as disease pressure and food availability, which have been shown to be important in other studies [7], [9], [18], [37], [60], could provide a plausible explanation for our findings, regarding variation in immune activities over both time and space.

### External factors

#### Effects of disease pressure

Risks of disease transmission and parasitic infections increase with animal density [61]–[67]; therefore, it was expected that the heavily-grazed goose moulting grounds [25], [68], [69] would be increasingly infested by parasites and pathogens. Indeed, the proportion of heterophils increased over time (Fig. 2C). Heterophils defend against extracellular pathogens by phagocytosis, and circulate in the blood ready to migrate to tissue during early stages of inflammation [54]. Similarly, the density of leukocytes showed an increase after an initial drop, and it seems plausible that the increase was a response to an increasing disease pressure. This interpretation is supported by Buehler et al. [16] who showed increased levels of leukocytes at stop-over sites where infection risk is likely to be elevated.

The increase of the H/L-ratio, indicating growing stress, may likewise have been caused by an increased disease pressure. The trend may have been exacerbated by stress directly or indirectly associated with moulting (inability to fly, increased vulnerability to predation) [66].

#### Effects of food availability

Typically, food availability on goose moulting grounds is highest halfway through the moulting season just before the most attractive foraging areas become depleted [25], [69]. Changes in density of leukocytes and lytic activity showed a striking similarity with the shifts in food resources with turning points of the trends occurring at similar dates (end of July). A possible causal link is supported by a study showing a close relationship between foraging rates and immune activities in young serins (*Serinus serinus*) [70]. Our observations suggest that lysis baseline levels increase as long as food resources allow (possibly to cope with increasing infection pressure) and drop when food is depleted. Density of leukocytes (Fig. 2A) appears complementary to lysis, as it decreased when lysis was increasing, and vice versa. This interpretation is in line with observations by Buehler et al. [37] who suggested that heterophils and lysis work together within the same costly “immune strategy”.

#### Effects of habitat

Comparing the temperate population with the Arctic population, we found differences in several of the investigated measures. Before discussing the immunological implications, we need to address several possibly confounding factors.

1. During acute stress, such as during catching and subsequent handling, leukocyte profiles in the blood may shift when heterophils move towards the peripheral blood while lymphocytes are redeployed to the lymph system [50], [71]. In the Arctic population, this stress-factor was apparent from the effect of “order of handling” on the relative abundance of lymphocytes and heterophils (decreasing and increasing, respectively, with order of handling, Table 1). In the temperate population, a similar stress effect could not be shown (slopes were 0.004±SE 0.003 and −0.001±SE 0.003 for proportions of lymphocytes and heterophils regressed against order of handling, respectively). We cannot exclude, therefore, the possibility that results have been affected by a difference in stress response by the two populations. However, this concerns only proportions of lymphocytes and heterophils and the H/L-ratio, as other measures did not exhibit an order of handling effect (Table 1).
2. Lytic activities of stored blood are affected by the quality of storage ([72], but see [73], [74]). As equivalent equipment was used at both locations and samples were kept frozen during transport, we feel that any difference in lytic activities between the populations is unlikely to result from differences in sample conservation. Similarly, differential leukocyte counts were not likely affected by transport. Blood smears from both locations were transported in a dry condition to the Netherlands and subsequently stained there.
3. The Arctic and temperate study areas differ in various (known or unknown) aspects. As in correlative studies in general, caution is needed when drawing any conclusions. Below we discuss proximate external factors that differ between the studied habitats, and which could have had an effect on immune activity: food availability and disease load.

The observed difference in immune activities between the Arctic and temperate population corresponds to previous studies that suggested that investment in immune defence is habitat-dependent, and well reflected by baseline immune activity [7], [9], [10], [16], [75]. First, foraging conditions vary widely between moulting sites in the high-Arctic and at temperate latitudes; peak plant standing crop (as a measure of goose food availability) in the Arctic is at most half of that found at temperate sites (data for the Netherlands in [76], for Svalbard in [77]), whereas food quality is similar [25]. The more favourable feeding conditions in the temperate habitat may have enabled the geese to invest more in immune activities [70]. Measures that we supposed to be affected by food conditions in the Arctic (see above) showed elevated values in the temperate area. Thus, factors important in shaping trends in baseline immunity within one area seem also effective in explaining differences between areas.

Another reason for the differences between the populations may be higher disease pressure, including the likelihood of parasitic infestations, in the temperate than in the Arctic population. The prolonged habitat use in the Netherlands allows parasites with environmental transmission mode, i.e. many helminths, ectoparasites and microbial pathogens with faecal-oral transmission, to locally accumulate as birds stay in the same habitat for the entire year. This leads to not only a higher parasite load but can result in evolution of more virulent and pathogenic strains (reviewed in [78]). The overall higher levels of immune activity, especially proportion eos+monoc and proportion reactive leukocytes, could reflect higher exposure to parasites in the temperate population.

Horrocks et al. [10] showed that in habitats where disease risk and humidity are low, lysis and agglutination titres are low as well. Our results confirm lower lysis titres in habitats where we expect a lower disease risk, though agglutination did not differ. This lack of difference in agglutination titres in our study is consistent with the results of Buehler et al. [16], [37], [79]. While lysis reflects the lysing of foreign blood cells as a result of complement action, hemagglutination indicates the action of natural antibodies [39]. Natural antibodies are related to adaptive immunity but differ from specific antibodies in their wide specificity and in that they are present without previous stimuli [66]. As adaptive defence is suggested to be cheaper in maintenance than innate defences (reviewed in [80]), this could be a reason why natural antibody titres are still reasonably high in the Arctic population.

### Internal factors

#### Moult

The proportion of eos+monoc was the only immune measure that exhibited a significant association with moult stage. The negative relationship (highest levels during early moult) could be due to the open, easily infected wound caused by the new, emerging feathers [43], which require increased levels of phagocytes for healing [81]. Surprisingly, there was only little support for the hypothesis that proportions of reactive leukocytes were influenced by moult stage (S3 Table). These cells could play a role to suppress dermal inflammation resulting from breaking of feather follicles but external (date) effects apparently obscured any moult-related activity.

#### Reproductive investment

Initiation of moult can be used as an indication of breeding history, as non-breeding and early-failed birds initiate moult earlier than breeding birds [56]. However, we did not have an independent measure of timing as we calculated initiation of moult from date and moult stage. Preliminary analyses (by testing effects of each time factor separately) indicated that moult initiation date did not perform better as an independent variable for variation in any of the immune measures than date or moult stage. Therefore, we found no evidence to suggest that previous breeding history affected immune activities as established for various bird species [82]–[85]. However, we found differences in immune measures between the sexes. Males had lower proportions of lymphocytes, higher proportions of heterophils, and consequently higher H/L-ratios but lower leukocyte densities. This indicates that males suffered higher stress-levels [49], [55] than females. This is possibly explained by their more vigilant behaviour [25]. Moreover, males spend less time feeding than females, which may result in less energy available to invest in immune functions.

### Conclusions

This is the first immune-ecological study on a wild Arctic population using multiple sampling occasions during a single life-history stage, the moult stage. We report variation in multiple indices of baseline immune activity over the moulting period, which was mainly related to date and, to a smaller degree, stage of moult. Date likely reflects changes in environmental factors, including disease pressure and food availability. The observed lower immune activity in the Arctic population of Barnacle geese compared to the temperate breeding population suggested that lower baseline levels are associated with lower disease risk. An intriguing next step would be a comparative study within the Arctic to further investigate the effect of variation in environmental factors, due to latitude and/or human impact, on immune activity during moult.

## Supporting Information

S1 Figure **Boxplots of each immune measure in the Arctic population.** Boxes represent data between the 25^th^ and 75^th^ percentiles. Thick bars inside the box indicate the median values. Whiskers indicate the 1.5 interquartile range. Outliers are indicated by circles.
(PDF)

S1 Table **Descriptive statistics for dependent and independent variables.** Descriptive statistics include sample size (N), mean, standard deviation (SD) for both dependent and independent variables, with additional maximum and minimum ranges for independent variables. Corresponding calendar dates for Julian dates 205 and 218 are 23 July and 5 August, respectively.
(DOCX)

S2 Table **Correlation matrices based on Pearson correlations.** Correlation matrices based on Pearson correlations for dependent and independent variables. Sample sizes of pairwise correlations are given for the dependent variables; sample sizes for independent variables are 338.
(DOCX)

S3 Table **Ranking of candidate models using Akaike Information Criterion (AIC).** Candidate models to explore effects of various independent variables on immune measures. Independent variables were: JD  =  Julian date, JD_SQ  =  Julian date squared, MS  =  moult stage, S  =  sex (male or female), Y  =  year (2007 or 2008), O  =  order of sampling. Models are listed when within 2 AIC units from the top-ranking model. Candidate models included all possible combinations of the independent variables (without interactions). K  =  number of parameters, Delta_AICc  =  difference in AICc with the top-ranking model, AICcWt  =  model weight, LL  =  log-likelihood.
(DOCX)
