# Supplementary figures and images for: Baseline Immune Activity Is Associated with Date Rather than with Moult Stage in the Arctic-Breeding Barnacle Goose (Branta leucopsis)
Source: PLoS One. 2014 Dec 17;9(12):e114812. doi: 10.1371/journal.pone.0114812 (PMC4269420; doi:10.1371/journal.pone.0114812)

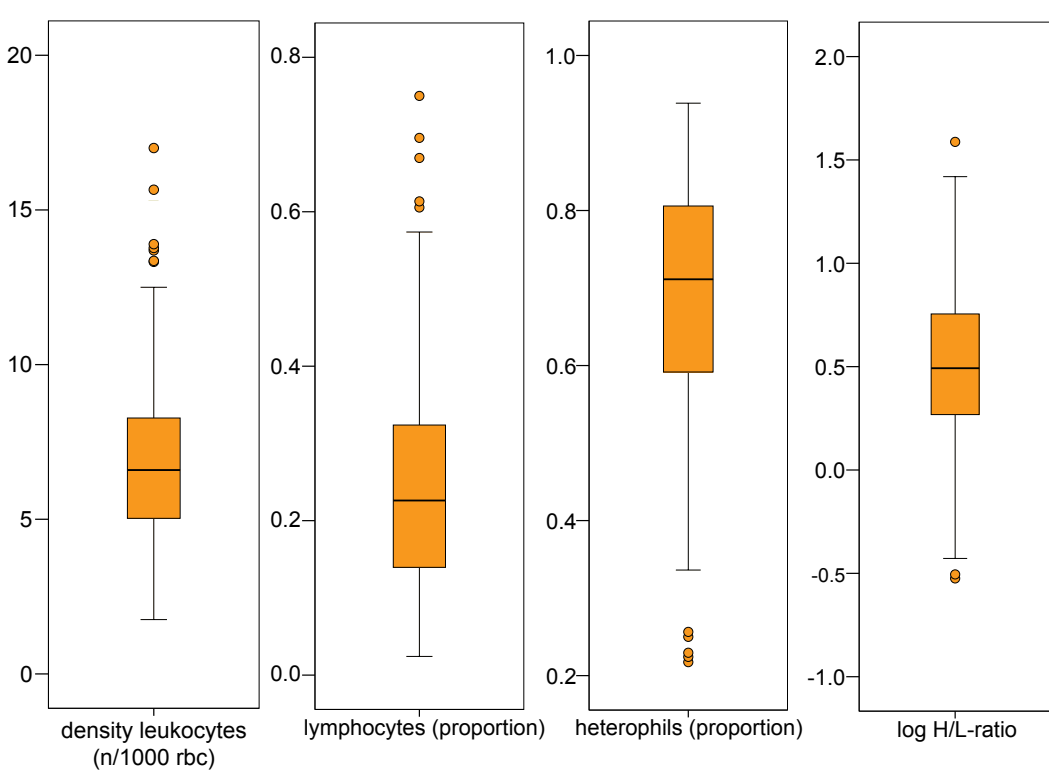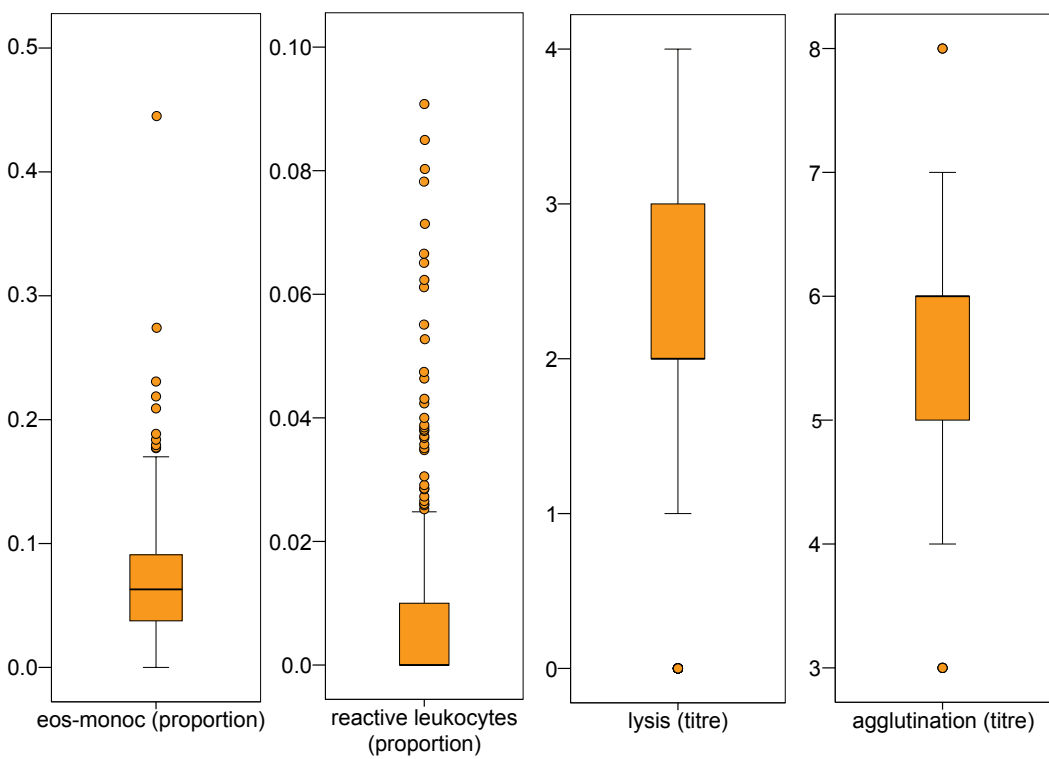

Supplement: S1 Figure — Boxplots of each immune measure in the Arctic population. Boxes represent data between the 25th and 75th percentiles. Thick bars inside the box indicate the median values. Whiskers indicate the 1.5 interquartile range. Outliers are indicated by circles. (PDF) [file pone.0114812.s001.pdf]
