# Supplementary material for: Baseline Immune Activity Is Associated with Date Rather than with Moult Stage in the Arctic-Breeding Barnacle Goose (Branta leucopsis)
Source: PLoS One. 2014 Dec 17;9(12):e114812. doi: 10.1371/journal.pone.0114812 (PMC4269420; doi:10.1371/journal.pone.0114812)
Supplement: S1 Table — Descriptive statistics for dependent and independent variables. Descriptive statistics include sample size (N), mean, standard deviation (SD) for both dependent and independent variables, with additional maximum and minimum ranges for independent variables. Corresponding calendar dates for Julian dates 205 and 218 are 23 July and 5 August, respectively. (DOCX) [file pone.0114812.s002.docx]

**Table S1. Descriptive statistics for dependent and independent variables.** Descriptive statistics include sample size (N), mean, standard deviation (SD) for both dependent and independent variables, with additional maximum and minimum ranges for independent variables. Corresponding calendar dates for Julian dates 205 and 218 are 23 July and 5 August, respectively.

| **Independent variables** | | **N** | **Mean** | **SD** | **Minimum** | **Maximum** |
| --- | --- | --- | --- | --- | --- | --- |
|  | Julian date | 338 | 210.7 | 4.0 | 205 | 218 |
|  | Moult stage | 338 | 11.7 | 7.4 | 0 | 26 |
|  | Moult initiation | 338 | 199.1 | 7.5 | 182 | 213 |
|  | Order of sampling | 338 | 28.8 | 18.3 | 1 | 81 |
| **Dependent variables** | | **N** | **Mean** | **SD** |  |  |
|  | Log density leukocytes (n per 1000 rbc) | 199 | 0.801 | 0.175 |  |  |
|  | Lymphocytes (proportion) | 249 | 0.247 | 0.138 |  |  |
|  | Heterophils (proportion) | 249 | 0.681 | 0.153 |  |  |
|  | Log H/L-ratio | 249 | 0.502 | 0.374 |  |  |
|  | Eos+monoc (proportion) | 249 | 0.072 | 0.051 |  |  |
|  | Reactive leukocytes (proportion) | 249 | 0.010 | 0.017 |  |  |
|  | Lysis (titre) | 232 | 2.040 | 0.957 |  |  |
|  | Agglutination (titre) | 232 | 5.700 | 0.946 |  |  |
