# Supplementary material for: Baseline Immune Activity Is Associated with Date Rather than with Moult Stage in the Arctic-Breeding Barnacle Goose (Branta leucopsis)
Source: PLoS One. 2014 Dec 17;9(12):e114812. doi: 10.1371/journal.pone.0114812 (PMC4269420; doi:10.1371/journal.pone.0114812)
Supplement: S2 Table — Correlation matrices based on Pearson correlations. Correlation matrices based on Pearson correlations for dependent and independent variables. Sample sizes of pairwise correlations are given for the dependent variables; sample sizes for independent variables are 338. (DOCX) [file pone.0114812.s003.docx]

**Table S2. Correlation matrices based on Pearson correlations.** Correlation matrices based on Pearson correlations for dependent and independent variables. Sample sizes of pairwise correlations are given for the dependent variables; sample sizes for independent variables are 338.

| **Correlation matrix of independent variables** | |  |  | **(1)** | **(2)** | **(3)** | **(4)** | **(5)** | **(6)** |  |  |
| --- | --- | --- | --- | --- | --- | --- | --- | --- | --- | --- | --- |
| (1) | Julian date |  |  | 1 |  |  |  |  |  |  |  |
| (2) | Moult stage |  |  | 0.251 | 1 |  |  |  |  |  |  |
| (3) | Moult initiation |  |  | 0.287 | –0.856 | 1 |  |  |  |  |  |
| (4) | Order of sampling |  |  | –0.185 | 0.063 | –0.161 | 1 |  |  |  |  |
| (5) | Year |  |  | –0.137 | –0.042 | –0.032 | –0.183 | 1 |  |  |  |
| (6) | Sex |  |  | 0.041 | 0.009 | 0.012 | –0.023 | 0.046 | 1 |  |  |
| **Correlation matrix of dependent variables** | |  |  | **(1)** | **(2)** | **(3)** | **(4)** | **(5)** | **(6)** | **(7)** | **(8)** |
| (1) | Log density leukocytes (n per 1000 rbc) |  |  | 1 |  |  |  |  |  |  |  |
| (2) | Lymphocytes (proportion) |  |  | –0.263 | 1 |  |  |  |  |  |  |
| (3) | Heterophils (proportion) |  |  | 0.224 | –0.945 | 1 |  |  |  |  |  |
| (4) | Log H/L–ratio |  |  | 0.259 | –0.986 | 0.963 | 1 |  |  |  |  |
| (5) | Eos+monoc (proportion) |  |  | 0.014 | 0.133 | –0.437 | –0.231 | 1 |  |  |  |
| (6) | Reactive leukocytes (proportion) |  |  | –0.12 | 0.099 | –0.072 | –0.11 | –0.025 | 1 |  |  |
| (7) | Lysis (titre) |  |  | –0.187 | 0.073 | –0.109 | –0.09 | 0.146 | –0.204 | 1 |  |
| (8) | Agglutination (titre) |  |  | 0.081 | –0.154 | 0.168 | 0.169 | –0.117 | –0.138 | 0.115 | 1 |
| **Sample sizes of (pairwise) correlations** | |  |  | **(1)** | **(2)** | **(3)** | **(4)** | **(5)** | **(6)** | **(7)** | **(8)** |
| (1) | Log density leukocytes (n per 1000 rbc) |  |  | 199 |  |  |  |  |  |  |  |
| (2) | Lymphocytes (proportion) |  |  | 196 | 249 |  |  |  |  |  |  |
| (3) | Heterophils (proportion) |  |  | 196 | 249 | 249 |  |  |  |  |  |
| (4) | Log H/L–ratio |  |  | 196 | 249 | 249 | 249 |  |  |  |  |
| (5) | Eos+monoc (proportion) |  |  | 196 | 249 | 249 | 249 | 249 |  |  |  |
| (6) | Reactive leukocytes (proportion) |  |  | 196 | 249 | 249 | 249 | 249 | 249 |  |  |
| (7) | Lysis (titre) |  |  | 139 | 174 | 174 | 174 | 174 | 174 | 232 |  |
| (8) | Agglutination (titre) |  |  | 139 | 174 | 174 | 174 | 174 | 174 | 232 | 232 |
