# Supplementary material for: Baseline Immune Activity Is Associated with Date Rather than with Moult Stage in the Arctic-Breeding Barnacle Goose (Branta leucopsis)
Source: PLoS One. 2014 Dec 17;9(12):e114812. doi: 10.1371/journal.pone.0114812 (PMC4269420; doi:10.1371/journal.pone.0114812)
Supplement: S3 Table — Ranking of candidate models using Akaike Information Criterion (AIC). Candidate models to explore effects of various independent variables on immune measures. Independent variables were: JD = Julian date, JD_SQ = Julian date squared, MS = moult stage, S = sex (male or female), Y = year (2007 or 2008), O = order of sampling. Models are listed when within 2 AIC units from the top-ranking model. Candidate models included all possible combinations of the independent variables (without interactions). K = number of parameters, Delta_AICc = difference in AICc with the top-ranking model, AICcWt = model weight, LL = log-likelihood. (DOCX) [file pone.0114812.s004.docx]

**Table S3. Ranking of candidate models using Akaike Information Criterion (AIC).** Candidate models to explore effects of various independent variables on immune measures. Independent variables were: JD = Julian date, JD_SQ = Julian date squared, MS = moult stage, S = sex (male or female), Y = year (2007 or 2008), O = order of sampling. Models are listed when within 2 AIC units from the top-ranking model. Candidate models included all possible combinations of the independent variables (without interactions). K = number of parameters, Delta_AICc = difference in AICc with the top-ranking model, AICcWt = model weight, LL = log-likelihood..

| **Immune measure** | **Model** | **K** | **AICc** | **Delta_AICc** | **AICcWt** | **LL** |
| --- | --- | --- | --- | --- | --- | --- |
| Density leucocytes (n/1000 rbc) | |  |  |  |  |  |
|  | JD+JD_SQ+Y+S+O | 7 | –142.26 | 0 | 0.25 | 78.42 |
|  | JD+JD_SQ+Y+S | 6 | –142.04 | 0.21 | 0.23 | 77.24 |
|  | JD+JD_SQ+MS+Y+S+O | 8 | –140.41 | 1.85 | 0.10 | 78.58 |
| Lymphocytes (proportion) | |  |  |  |  |  |
|  | JD+S+O | 5 | 126.97 | 0 | 0.33 | –58.36 |
|  | JD+JD_SQ+S+O | 6 | 128.46 | 1.49 | 0.16 | –58.06 |
|  | JD+Y+S+O | 6 | 128.81 | 1.85 | 0.13 | –58.23 |
| Heterophils (proportion) |  |  |  |  |  |  |
|  | JD+S+O | 5 | 133.10 | 0 | 0.31 | –61.42 |
|  | JD+MS+S+O | 6 | 134.22 | 1.12 | 0.18 | –60.94 |
|  | JD+Y+S+O | 6 | 134.98 | 1.89 | 0.12 | –61.32 |
| H/L-ratio |  |  |  |  |  |  |
|  | JD+S+O | 5 | 195.44 | 0 | 0.32 | –92.60 |
|  | JD+JD_SQ+S+O | 6 | 196.91 | 1.48 | 0.15 | –92.28 |
|  | JD+Y+S+O | 6 | 197.39 | 1.95 | 0.12 | –92.52 |
| Eos+monoc (proportion) | |  |  |  |  |  |
|  | JD+JD_SQ+MS+Y | 6 | –151.89 | 0 | 0.15 | 82.12 |
|  | MS+Y | 4 | –151.59 | 0.30 | 0.13 | 79.88 |
|  | JD+JD_SQ+MS+Y+O | 7 | –151.16 | 0.74 | 0.10 | 82.81 |
|  | MS+Y+O | 5 | –150.59 | 1.31 | 0.08 | 80.42 |
|  | MS+Y+S | 5 | –150.50 | 1.39 | 0.08 | 80.37 |
|  | JD+JD_SQ+MS+Y+S | 7 | –150.35 | 1.54 | 0.07 | 82.41 |
|  | JD+MS+Y | 5 | –150.10 | 1.79 | 0.06 | 80.17 |
| Reactive leukocytes (proportion) | |  |  |  |  |  |
|  | JD+JD_SQ+Y+O | 11 | 590.68 | 0 | 0.32 | –283.57 |
|  | JD+JD_SQ+Y | 9 | 591.62 | 0.95 | 0.20 | –286.36 |
|  | JD+JD_SQ+MS+Y+O | 13 | 591.98 | 1.31 | 0.17 | –281.78 |
| Lysis (titre) |  |  |  |  |  |  |
|  | JD+JD_SQ+Y | 4 | 676.85 | 0 | 0.33 | –334.34 |
|  | JD+JD_SQ+Y+S | 5 | 677.89 | 1.04 | 0.20 | –333.81 |
| Agglutination (titre) | |  |  |  |  |  |
|  | JD+JD_SQ | 3 | 871.75 | 0 | 0.07 | -432.82 |
|  | O | 2 | 872.06 | 0.30 | 0.06 | -434.00 |
|  | JD+JD_SQ+O | 4 | 872.40 | 0.65 | 0.05 | -432.11 |
|  | Y | 2 | 872.41 | 0.65 | 0.05 | -434.18 |
|  | JD+Y | 3 | 872.65 | 0.90 | 0.05 | -433.27 |
|  | Y+O | 3 | 872.67 | 0.92 | 0.05 | -433.28 |
|  | JD+JD_SQ+Y | 4 | 872.81 | 1.06 | 0.04 | -432.32 |
|  | JD+O | 3 | 873.19 | 1.44 | 0.04 | -433.54 |
|  | MS+O | 3 | 873.31 | 1.55 | 0.03 | -433.60 |
|  | JD | 2 | 873.55 | 1.80 | 0.03 | -434.75 |
|  | JD+Y+O | 4 | 873.63 | 1.88 | 0.03 | -432.73 |
